# Low rate of surgery in juvenile idiopathic scoliosis treated with a complete and tailored conservative approach: end-growth results from a retrospective cohort

**DOI:** 10.1186/1748-7161-9-12

**Published:** 2014-08-18

**Authors:** Claudia Fusco, Sabrina Donzelli, Monia Lusini, Minnella Salvatore, Fabio Zaina, Stefano Negrini

**Affiliations:** 1 ISICO (Italian Scientific Spine Institute), Milan, Italy; 2 Physical and Rehabilitation Medicine, Department of Clinical and Experimental Sciences, University of Brescia, Brescia, Italy; 3 IRCCS Don Gnocchi Foundation, Milan, Italy

## Abstract

**Background context:**

The main distinctive aspect of Juvenile Idiopathic Scoliosis (JIS) with respect to Adolescent Idiopathic Scoliosis (AIS) is the high risk of severe deformity and surgery. Approximately 70% of curves in patients with JIS progress and ultimately require surgery. There are presently very few studies with long-term follow-up of JIS and even fewer looking specifically at bracing Purpose To verify the effectiveness of a complete conservative treatment, including bracing and exercises, for JIS.

**Study design/setting:**

Retrospective cohort observational study nested in a clinical prospective database of consecutive outpatients. Patient Sample Inclusion criteria: JIS, no previous treatment, all consecutive radiographies available from treatment start to end of growth (Risser sign 3). We found 30 patients, 27 females, 10 JIS type 1; mean age at first diagnosis was 7.8 +/-1.5 and mean treatment lasted 5.8 years. Cobb degrees 24.4+/-10 degrees, with 7 cases >30 degrees, and 2 > 45degrees. Outcome Measures Physiological measures. Radiographic and clinical data.

**Methods:**

Treatment (exercises alone, or elastic-rigid-highly rigid braces plus exercises) was tailored and continuously changed according to Cobb degrees, individual preferences, anthropometric characteristics, pubertal spurt, remaining growth, rotation, hump, lumbar curve take-off, and imbalance. The SOSORT Guidelines for patients’ management have been followed. Funding and Conflict of Interest: no.

**Results:**

33.3% (95% Confidence Interval 16.4-50.2%) of patients worsened over the years. At the end of growth, 6.6% (0–15.5%) had surgical deformities (>45degrees). We observed a good correction in the first years of treatment until pubertal growth spurt, when progression was usually noted and treatment changed increasing corrective forces (hours or rigidity of bracing). 23 cases were followed up until they had two consecutive radiographies showing Risser sign 5 and showed stability.

**Conclusions:**

Conservative treatment initiated already in childhood may favorably change the natural history of JIS with the aim of reaching a curve as far as possible from surgical thresholds. Observation, physical exercises, braces can be useful tools in the hand of physicians, but they must be carefully utilized by a deep knowledge of JIS.

## Background

According to the SRS (Scoliosis Research Society) Juvenile idiopathic scoliosis (JIS) [1] is defined as a scoliosis that is first diagnosed between the ages of three years and ten years. It is further classified on the basis of the age of the patient at first identification of the deformity as either “juvenile 1” for children between the ages of 3 and 7, or “juvenile 2” between 8 and 10 years.

Since spinal growth is fairly steady during this juvenile period, Dickson and Archer [2] believed that true juvenile-onset scoliosis was rare enough not to warrant a separate category. For this reason, they proposed a two-group classification that included early onset (five years of age or less) and late onset (six years of age and older) scoliosis. According to this proposal, patients who receive a diagnosis of scoliosis at five years of age or younger have a much higher chance of having a large curve develop, which may lead to pulmonary complications and cor pulmonale.

In JIS the same prevalence has been shown in the two genders in children between three and six years of age, with a gradual increase in females until ten years, when the ratio males/females become close to that reported for patients with adolescent idiopathic scoliosis (AIS).

The natural history of JIS is characterized by an early deformity that leads to a major but extremely variable risk of progression throughout the pubertal growth spurt.

The main distinctive aspect of JIS with respect to AIS is that, since the curves occur at such a young age, there is a higher risk of severe deformity for these patients than for patients with AIS. Approximately 70% of curves in patients with JIS progress and ultimately require surgery [3]. Data in the literature confirm this progressive trend of JIS and also its rapid worsening in puberty requiring surgical treatment: Tolo and Gillespie [4] in a case series of patients with an ample range of curves (from less than 20° to more than 50°) reported progression in forty-two out of fifty-nine curves (71%), sixteen of which required surgery. Similarly, Figueiredo and James [5] reported that fifty-five out of ninety-eight patients (56%) with JIS of 43° on average required spinal surgery after a period of three years on average of bracing (Edinburgh brace).

Despite this major aggressiveness, there are presently very few studies with long-term follow-up of JIS and even fewer looking specifically at bracing in this group [6]. One of the few studies, by Robinson and McMaster [7] in 1996, followed 89 of 109 JIS patients to skeletal maturity. Patients (47° on average) were braced full-time or part-time (20 hours/day) with a Milwaukee or Boston brace, and 67% of them progressed to spinal fusion. Jarvis investigated the effectiveness of part-time bracing (Charleston nighttime bending brace) for JIS of 30° with patients followed to skeletal maturity. He reported that 51% were successfully managed with the brace, better than the natural history [8]. In a recent study Aulisa observed 113 patients for a minimal follow up of 24 months and in agreement with SRS criteria of outcome he reported a curve correction in 77.8% of patients, a stabilization in 15.9% of them and a progression in 6.19% by using different type of brace .

The aim of this study is to verify the effectiveness of a custom-made complete conservative treatment, including bracing and exercises, for JIS.

## Material and methods

### Study design

We performed a retrospective cohort observational study nested in a clinical prospective database of consecutive outpatients started in March 2003. The study was performed on the 31st of December 2009. We included all consecutive patients in the database who reached the End of Growth, including data on some patients that started treatment before database inception. This was a clinical everyday practice study in which the physicians were neutral observers because they were not aware of the study being performed and were focused only on the children’ needs. All patients were evaluated by the same physicians according to the same clinical and radiological criteria. As radiographic parameter of skeletal maturity we considered the European Risser staging 3 (corresponding to American Risser 4) [9,10].

### Population

The inclusion criteria were: JIS, no previous treatment, all consecutive radiographies available from treatment start to Risser sign 3, last evaluation before December 31st 2009. Exclusion criteria were: secondary scoliosis and pathologies known to be possible causes of scoliosis, neurological deficits, a difference in inferior limb length exceeding 10 mm, previous treatment for scoliosis (brace, exercises or surgery) and scoliosis onset before three years or above 10 years of age. We found 30 patients (Table 1), 27 females and 3 males, 10 with a diagnosis of JIS type 1 and 20 with type 2; mean age at first diagnosis was 7.8 ± 1.5 and mean treatment lasted 5.8 years. 4 curves were thoracic, 24 were thoracic and lumbar and 2 curves were thoracolumbar. The mean Cobb angle at the beginning of treatment was of 24.4° Cobb for the thoracic curves, 17° Cobb for the thoracolumbar curve and 22.4 for thoracic one (Table 2) . All patients were evaluated every 6 months until the European Risser sign 3 that was considered in the statistical analysis as the skeletal maturity; in particular we observed 23 patients in a long term follow up until Risser sign 5. Finally, we split the patients into 3 groups according to degree of curvature at first diagnosis: 9 patients presented a curve under 20°, 11 patients between 20 and 30° and 7 were over 30°.

**Table 1:** The table shows general data of the population

**Gender**	**Type of curve**	**JIS type**
27 Females	4 Thoracic	10 Type 1
3 Male	24 Thoracic and lumbar 2 Thoraco-lumbar	20 Type 2

**Table 2:** The table summarized the clinical results obtained

	**Thoracic curve**		**Thoracolumbar curve**		**Lumbar curve**	
	*Start*	*End*	*Start*	*End*	*Start*	*End*
**Cobb degrees**	24.4	25.8	17.0	15.8	22.4	22.5
**Bunnel degrees**	8.2	13.2	0.5	0.3	1.7	1.7
	**Patients**					
	*Start*	*End*				
**TRACE index**	2.2	3.4				

For Cobb and Bunnel degrees we did not find statistically significant differences between start and end of treatment; contrarily, aesthetic (TRACE index) difference was statistically significant (P < 0.05).

We were able to collect the clinical history of each patient from childhood to adolescence, and for 23 patients until almost 19 years of age.

### Treatment

Treatment was tailored according to individual preferences, anthropometric characteristics and other risk factors such as pubertal spurt, remaining growth, rotation, hump, lumbar curve take-off, and imbalance. From this perspective, 2 patients were only treated with physiotherapeutic specific exercises until the end of treatment; 26 with just a brace from the start of treatment; 2 patients started treatment with specific exercises and afterward added bracing at their pubertal growth spurt; 4 patients among those who began treatment with brace changed brace type to a more corrective brace during puberty. The SOSORT Guidelines for patients’ management have been strictly followed [11,12].

In order of therapeutic efficacy (in our view, from the least strong to the strongest) [11,12], the braces that were considered in this study are: Spinecor [13-15], Sibilla [16], Lyon [17] and Sforzesco braces [16,18,19]. The first was prescribed for children with a curvature less than 30° that could not be controlled through SEAS exercises alone. It is a dynamic brace that allows correction of spinal curvature while retaining great freedom of movement for young patients. The Sibilla brace is the less rigid brace we use in our Institute: we almost always choose this brace in children, due to the reduced forces necessary to possibly reach a stable curve [20]. In the past we used the Lyon brace for major curves, often in cases of worsening during the pubertal spurt, but from 2003 onward we replaced it with the Sforzesco brace, which is the most rigid brace we use today [21]. Bracing was fully customized to individual needs. In general, we started the brace wearing at Risser sign 0 in a range of Cobb degrees between 20 and 30 according a series of risk factors like rapid growth, curve stiffness, aesthetic index. The wearing hours we prescribed were between to 18 hours per day to 23 hours per day until the skeletal maturity. As soon as Risser sign 3 was reached, a progressive weaning was started reducing brace prescription below 18 hours per day. The reduction was of 2 hours every 6 months according to the stability of the curve and to the correction reached.

Sport was prescribed for all patients and, from 7–8 years of age, children were also able to perform physiotherapeutic specific exercises (PSEs) [11,22,23]. We prescribed exercises according to the SEAS approach based on active self-correction, spinal stabilization and development of balance reactions and neuromotor integration [24-27] .

### Outcome criteria and statistics

Data included history, scoliosis clinical parameters (ATR and rib hump, and TRACE for aesthetics) [28-31], sagittal profile [32,33] and growth parameters (height, weight, menarche), and radiographic data (Cobb degrees and Risser sign). With regard to outcome criteria, we considered the modification of the ATR, the rib hump, and the TRACE as clinical criteria; as radiographic criteria we evaluated variations in Cobb degrees, and considered 5° as the cut-off value for worsening. We compared clinical and radiographic variations at the first evaluation (Risser sign 0) and at Risser sign 3 to assess the effect of conservative treatment on JIS. For statistical analysis, after checking for normal distribution, we performed T-tests with Bonferroni correction. We also calculated 95% Confidence Interval for proportions (95IC).

All patients and their parents accepted data management for research purposes; due to the observational setting, ethical approval was not required.

## Results

33.3% (95IC 16.4-50.2%) of patients worsened over the years. The percentage of curves arriving at the surgical threshold, considered to be 45°, was 6.6% (95IC 0–15.5%) and it is represented by two patients that at first evaluation (Risser sign 0) presented dorsal curves of 47° and 51°, respectively, and arrived at Risser sign 4 with 49° and 46°.

**Figure 1:**
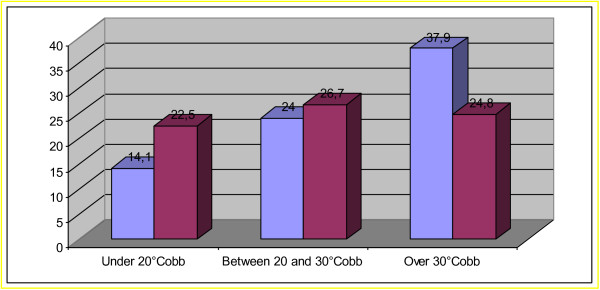
Under 20 Cobb degrees and between 20 to 30 Cobb degrees group the graphic shows a consistent but not significant worsening; Over 30° group: not significant improvement.

At Risser 3 (Table 2) we observed not statistically significant differences in terms of average Cobb degrees respect the beginning of treatment (25.8° Cobb for thoracic curve, 15.8° Cobb for thoracolumbar and 22.5° Cobb for lumbar curve). To analyze the importance of curve amplitude at the beginning of treatment, we observed the evolution of curves clustered in three subgroup and noted a consistent but statistically not significant change in the major curve in the subset below 20°, light worsening in the subset between 20° and 30°, and a statistically not significant improvement from 37.9 ± 7.9 to 24.8 ± 6.1 in the group of patients who started treatment above 30° (Figure 1).

We collected 23 cases that were followed for a very long period until they reached the end of growth (two consecutive radiographies showing Risser sign 5). We compared curvature at Risser sign 3 and at Risser sign 5 and observed good maintenance of results reached at Risser sign 3, with a not statistically significant change from 28.3 ± 10.1 to 28.7 ± 9.7 in thoracic curves and from 17.6 ± 12.1 to 18.1 ± 12.2 in lumbar curves. Seven out of 23 patients (30.4%) arrived at the end of treatment with a curve above 35° and 2 out of 23, the same patients identified in the previous analysis (8.7% in this sub-sample), presented a curve above 45°.

We did not find statistically significant differences according to scoliosis topography. We also observed a better evolution in lumbar and thoraco-lumbar humps than in thoracic ones, with a statistically not significant worsening of Bunnell degrees from 8.2 ± 9.0 to 13.2 ± 2.2, and of hump height from 2.2 ± 4.9 mm, to 6.5 ± 3.8 mm.

The most impaired clinical finding was aesthetics, possibly due to the growth of the child. In fact with regard to TRACE we observed a statistically significant worsening from 2.2 ± 2.1 pre-treatment to 3.4 ± 1.4 post-treatment.

## Discussion

According to our knowledge, this is the first study about a complete conservative treatment including bracing and exercises in JIS. It shows progression in 33.3% of cases, with curves ending above 45° in 2 cases (6.6%): both cases were above this value at start of treatment, and remained stable throughout growth.

According to the literature, also in our sample JIS presents an evident worsening during the growth spurt. [34] Twenty-eight out of 30 patients worsened in that period, when an increase in strength of treatment was adopted, represented by a passage from physical exercises only to brace, or changing of the brace to one more rigid, or increased hours of brace wearing. This could be one of the reasons of our results: the readiness to reassess initial choices against others which are more demanding at the start of puberty.

Despite current literature data, we observed that conservative treatment can overcome this natural worsening trend of JIS. In our study, the rate of patients who arrived at the end of growth with a curve above 45° is much less than that reported in other studies [3,4]. This may be due to our setting, focused on conservative treatment: every single patient represents a challenge to try to avoid surgery. With this goal in mind, treatment is tailored for each young patient with an eye to the present and an eye to the future. This means that we know the very high likelihood of worsening of JIS and we propose a strong treatment from the beginning, even if this leads to prescribing a brace for a child. We avoid waiting and seeing what will happen in adolescence, but start to face scoliosis from childhood. This behavior is recently confirmed also in the current literature [35]: JIS surgeons with more experience were less likely to opt for definitive fusion. Moreover primary care providers play an important role in the early diagnosis by observation of the young patients and in the therapeutic decision making [36]. In this field is remarkable the importance of scoliosis screening as a tool for first diagnosis and also for observation made by the specialist that may be considered the first step of scoliosis treatment.

In an overall analysis, we observed a good corrective power of braces in the first years of treatment until pubertal growth spurt: this caused a decrease in hours of brace wearing in the years before puberty, with maintenance of the achieved correction. This report is in agreement with data in the literature and the every day observation that JIS can be better corrected in the pre-adolescent period [34]; the most dangerous period for worsening is the growth spurt, while the final period, until the end of growth, in our cohort is characterized by maintenance of results previously obtained or, rarely, by slight worsening while gradually weaning the brace. In this context Aulisa demonstrated the effectiveness of brace treatment in JIS: the brace appears to be more effective with curves under 30° Cobb degrees than curves over 30°.

Two patients arrived at the surgical threshold but, upon careful analysis, they had a critical medical case from the beginning, indeed both started treatment with a major curve, already considered surgical; in our view (and in that of the parents, that chose this approach) the use of a brace plus specific physiotherapeutic exercises allowed in these patients to avoid many surgical interventions and further worsening.The results obtained from clustering patients in three groups according to Cobb degrees led to a conclusion that may be obvious: JIS that at the beginning are light have a major potential to progress (Figure 1). The practical relapsing of this finding in everyday clinical practice may be that we need to be “afraid of scoliosis” from the very beginning, in order to acquire careful behavior independent from the number of Cobb degrees; young age is still a risk factor. It is important to choose the best treatment in childhood in order to arrive at the doorway of adolescence with a smaller hump entity and as few Cobb degrees as possible, so to be ready to face future worsening.

The final period, from Risser sign 3 to the end of growth, was demonstrated in this study to be characterized by a tendency to stability or slight worsening, even if bracing was continued and gradually weaned. This confirms the need to continue treatment until bone maturity.

The main limit of this study is its retrospective design: we have not a complete overview about all patients visited in that period so we could not consider patients that had dropped out. Two patient that arrived at Risser 5 with a surgical curve, in the moment of data analysis, were not yet fused. Further prospective studies are needed to investigate this particular kind of scoliosis and to observe corrective possibilities in JIS by a complete conservative approach.

## Conclusions

Conservative treatment initiated already in childhood may favorably change the natural history of JIS with the aim of reaching a curve as far as possible from surgical thresholds. Observation, physical exercises, braces can be useful tools in the hand of physicians, but they must be carefully utilized by a deep knowledge of JIS.

## Abbreviations

JIS: Juvenile Idiopathic Scoliosis; SRS: Scoliosis Research Society; AIS: Adolescent Idiopathic Scoliosis; ATR: Angle of Trunk Rotation; TRACE: Trunk Aesthetic Clinical Evaluation; SOSORT: International Society On Scoliosis Orthopaedic And Rehabilitation Treatment.

## Competing interests

The authors declare that they have no competing interest.

## Author’s contributions

All Authors contributed equally to this work, all Authors read and approved the final manuscript.
